# Editorial: Influence of social determinants on wellbeing in chronic kidney disease patients

**DOI:** 10.3389/fpubh.2025.1719473

**Published:** 2025-10-29

**Authors:** Evangelos C. Fradelos, Victoria Alikari

**Affiliations:** ^1^Department of Nursing, School of Health Science, University of Thessaly, Larissa, Greece; ^2^Department of Nursing, University of West Attica, Athens, Greece

**Keywords:** chronic kidney disease, hemodialysis, social determinants, wellbeing, quality of life

Chronic Kidney Disease (CKD) is a major global public health problem, with incidence and mortality increasing every year. According to Wang et al., between 1990 and 2021, CKD cases doubled, while age-standardized mortality (ASDR) and disability rates (ASDAR) increased in almost all countries, underscoring the continuing severity of the disease. Older adults and men exhibit higher ASDAR rates, with the greatest burden of CKD attributed to hypertension and type 2 diabetes. The global rise in CKD is linked to metabolic, environmental, and socioeconomic factors, while the lack of early diagnosis and preventive policies further exacerbates the condition. Identifying risk factors and implementing effective prevention strategies are therefore critical to reducing CKD-related mortality and disease burden.

CKD extends beyond a medical diagnosis, constituting a continuous biopsychosocial and deeply existential experience. Being diagnosed and undergoing hemodialysis treatment marks a transition from autonomy to dependence on treatment. According to Ramírez-Perdomo (1), individuals living with CKD experience variations and changes across multiple existential dimensions, including Relationality (feelings of entrapment, perception of terminality, need for support), Temporality (the unexpected nature of diagnosis, difficulty remaining present, experience of being young and ill), Corporeality (bodily deterioration, changes in sexual life), Materiality (financial impact), and Spatiality (changes in daily life, sadness, and depression). For these individuals, the essence of time is organized around the treatment cycle, and daily life is restructured around renal care. At this point, the common question most patients ask — “How do I go on?” — is transformed into “Who am I, living this way?”

According to O'Hare et al. (2), illness radically alters an individual's identity. Biological trauma is accompanied by an existential rupture, which gives rise to feelings of fear, anger, guilt, or futility, but can also serve as a catalyst for reflection and transformation. As theorists such as Parse (3), Frankl (4), and Ricoeur (5) argue, the experience of illness—if lived consciously and with accompaniment—can become a space of authenticity and meaning, where the individual rediscovers the value of existence, relationships, and gratitude. Dialysis, although medically defined, acquires an almost ritualistic character: an act of survival but also a constant reminder of fragility. Each session represents an encounter with limits—of the body, of time, of hope (6). The patient is called upon to redefine their relationship with themselves, with others, and with the world—a process that, while it may entail suffering, simultaneously opens the way toward a deeper understanding of what it means to live (7).

However, this journey does not occur in a vacuum. The experience of CKD is deeply shaped by social determinants of health (SDoH)—such as income, education, employment, housing, access to healthcare services, environmental conditions, social relationships, and cultural values—all of which influence both disease progression and the patient's ability to maintain quality of life and internal equilibrium (Li et al.). Poverty, social isolation, and unemployment can increase psychological distress and reduce adherence to treatment (Lian and Wang). Conversely, supportive social networks, cultural acceptance, and spirituality can serve as protective factors, enhancing resilience and meaning in life. CKD, therefore, is not merely a medical condition but also a reflection of social and existential inequalities (8, 9).

Our Research Topic, entitled “*The Impact of Social Determinants on the Well-being of Patients with Chronic Kidney Disease*,” launched in Frontiers, explores the influence of social determinants on the wellbeing, health, and quality of life of individuals living with CKD. Within this Research Topic, 10 articles have been published, which collectively have received more than 24,000 views, 17,000 reads, and 5,645 downloads to date. These articles address a wide range of themes, from the impact of social determinants of health on disease progression (Lalo et al.), to the relationship between environmental exposure, diagnosis, and disease trajectory (Liang and Li), and mental wellbeing (Beyrami and Amiri). This body of research investigates both the direct and indirect effects of social determinants on the wellbeing of individuals diagnosed with CKD, while also proposing causal mechanisms that link social inequalities and resource availability to disease outcomes. The findings can inform health policy development by supporting the design of evidence-based, patient- and workforce-centered interventions, emphasizing the need to strengthen access to healthcare services, provide psychosocial support, train professionals in existential care and communication, and acknowledge social inequalities as determinants of care (10).

In today's technocratic society, where care tends to become cold, distant, and impersonal, the existential dimension of nursing reminds us of the urgent need to redefine the meaning of health and illness within a human-centered and holistic framework. Hemodialysis may prolong life, yet it is meaning that renders life worth living. The care of individuals with Chronic Kidney Disease (CKD) should therefore focus not only on biological survival but also on the process of meaning-making, encompassing both the biological and existential dimensions of health and illness at individual and societal levels (11).

Within this context, technology emerges as an inseparable but ambivalent component of care. Nurses are required to combine technical proficiency with interpersonal and empathic competence, recognizing technology as an integral element of holistic, humanistic care—one that must never overshadow the patient. The hemodialysis setting exemplifies this complexity, as technology and nursing coexist and interact continuously. The patient and the machine become physically connected, yet they must remain distinct entities. As Barnard (14) argues, when a nurse identifies the sound of an alarm with the patient themself, critical questions arise: Is the machine an extension of the patient or of the nurse? Such perceptions may diminish the patient's image as a unique and autonomous person. Thus, nurses must cultivate self-awareness and reflexivity, ensuring that their behaviors and interactions with technology do not depersonalize care but rather enhance it.

Evidence suggests that in dialysis units, technical expertise and precision are highly valued, as they are directly linked to measurable outcomes—such as urea clearance, hemoglobin, and phosphorus levels—and confer professional status and authority within the clinical hierarchy. However, excessive emphasis on technical performance risks overshadowing the relational essence of care. The notion of existential care entails an authentic presence and accompaniment—a mode of “being with” the patient, in which their experience is acknowledged without attempts to correct, interpret, or rationalize it (12).

Consequently, the role of the nurse is fundamentally expanded: from a technical provider of care to a companion in the patient's search for meaning—a professional who bridges biomedical knowledge with existential understanding. Care thus becomes an act of participation, relationship, and meaning-making, in which technology supports, rather than supplants, human connection. True wellbeing is not defined by the absence of symptoms or the achievement of clinical targets, but by the presence of meaning, dignity, and social justice. Only then can care in the context of chronic kidney disease be considered genuinely holistic—embracing human existence in all its depth, transcending the boundaries of disease, and guiding the individual toward a path of authenticity, meaning, and solidarity (13).
